# Effects of Beetroot-Derived Nitrate Supplementation in Highly Trained and Elite Athletes: A Scoping Review of Randomized Controlled Trials on Performance, Recovery, and Physiological Outcomes

**DOI:** 10.3390/nu18152551

**Published:** 2026-08-04

**Authors:** Nafih Cherappurath, Muhammed Navaf, Mevlüt Yıldız, Halil İbrahim Ceylan, Muhammed Ali Thoompenthodi, Masilamani Elayaraja, Raul Ioan Muntean, Valentina Stefanica, Kappat Valiyapeediyekkal Sunooj, Muneer Pelasseri

**Affiliations:** 1 Department of Physical Education, Amal College of Advanced Studies (Autonomous), Nilambur 679329, Kerala, India; 2 Department of Agricultural Engineering, College of Agriculture, Kerala Agricultural University, Vellanikkara 680656, Kerala, India; 3 Coaching Sciences, Faculty of Sports Sciences, Mugla Sitki Kocman University, Mugla 48000, Türkiye; 4 Faculty of Sports Sciences, Atatürk University, Erzurum 25100, Türkiye; 5 Department of Physical Education, Malabar College of Advanced Studies, Vengara 676519, Kerala, India; 6 Department of Physical Education and Sports, Pondicherry University, Puducherry 605014, Puducherry, India; elaya.cricket@gmail.com; 7 Department of Physical Education and Sport, Faculty of Law and Social Sciences, University “1 Decembrie 1918” of Alba Iulia, 510009 Alba Iulia, Romania; 8 Department of Physical Education and Sport, Faculty of Sciences, Physical Education and Informatics, National University of Science and Technology Politehnica Bucharest, 110040 Pitesti, Romania; valentina.stefanica@upb.ro; 9 Department of Food Science and Technology, Pondicherry University, Puducherry 605014, Puducherry, India; sunooj4u@gmail.com; 10 Department of Physical Education, University of Calicut, Thenhipalam 673635, Kerala, India

**Keywords:** beetroot juice, dietary nitrate, nitric oxide, endurance exercise, anaerobic performance, ergogenic aids, exercise physiology, sports nutrition

## Abstract

**Background:** Beetroot-derived nitrate supplementation is a widely studied nutritional strategy to enhance athletic performance. Although ergogenic benefits have been reported in recreational and moderately trained individuals, evidence for highly trained and elite athletes remains inconsistent. **Objectives:** This scoping review aimed to map and synthesize evidence from randomized controlled trials (RCTs) investigating the effects of beetroot-derived nitrate supplementation on performance, recovery, and physiological outcomes in highly trained and elite athletes. **Methods:** The review followed the Joanna Briggs Institute methodology and PRISMA-ScR guidelines. Literature searches were conducted in PubMed, Scopus, and Web of Science. Eligible studies included RCTs involving McKay Tier 3–5 athletes and evaluating beetroot-derived nitrate supplementation as a standalone intervention. **Results:** Thirty-one RCTs met the inclusion criteria, representing athletes from a wide range of sports. Supplementation protocols varied substantially, with nitrate doses ranging from 4 to 19.5 mmol·day^−1^ (248–1209 mg·day^−1^) and durations from acute administration to 15 days. Positive effects were most frequently reported for cycling time-trial performance, Yo-Yo intermittent recovery performance, anaerobic power, neuromuscular performance, exercise tolerance, and recovery. In contrast, findings for endurance performance, sport-specific skills, oxygen consumption, and perceptual responses were inconsistent despite marked increases in nitrate and nitrite bioavailability. The outcomes seemed to depend on dosage, duration, sport type, and athletes’ training status. **Conclusions:** Beetroot-derived nitrate supplementation may enhance performance in highly trained and elite athletes, particularly during high-intensity and anaerobic activities. However, the effects are inconsistent, underscoring the need for further research to establish optimal supplementation strategies and evaluate long-term efficacy in elite sporting populations.

## 1. Introduction

Beetroot-derived nitrate supplementation has emerged as a prominent nutritional strategy in sport and exercise science for its potential to enhance athletic performance via nitric oxide (NO)-mediated mechanisms. The nitrate–nitrite–NO pathway plays a central role in this process, whereby dietary nitrate is reduced to nitrite and subsequently to nitric oxide, particularly under the hypoxic and acidic conditions typical of exercise [1,2,3]. This increase in NO bioavailability promotes vasodilation, improves blood flow, and enhances oxygen and nutrient delivery to working muscles, thereby improving exercise efficiency and performance. A recent study indicates that both acute and chronic intake of nitrate-rich beetroot juice can enhance endurance performance, with significant improvements in VO_2_max and reduced oxygen costs during submaximal exercise [4]. Additionally, beetroot juice may offer unique benefits beyond nitrate, such as antioxidant and anti-inflammatory properties, which could further aid recovery and performance adaptations [5]. Recent studies suggest that beetroot juice supplementation may improve muscular endurance, total exercise distance, peak power output, and sprint performance. However, these benefits are not consistent across all exercise outcomes or populations, with responses varying between healthy adults and trained athletes [6,7,8].

Evidence suggests that supplementation with beetroot-derived nitrates may enhance physical performance and cognitive function [1], increase resistance during repeated-sprint exercise [9], and provide ergogenic benefits during team-sport activities [10]. Moreover, benefits have been reported in strength and recovery, with improvements in maximal voluntary contraction and attenuation of force decline following exercise, particularly in combat and strength-based sports [11,12]. Enhancements in cardiorespiratory endurance, including improvements in $\dot{V}O_{2}\max$ and exercise efficiency, have also been observed, especially in endurance-based activities such as cycling and running [13,14].

Consequently, dietary nitrate supplementation has received considerable attention as a potential ergogenic aid for enhancing athletic performance across a range of sporting disciplines [15,16,17,18]. Proposed mechanisms include enhanced mitochondrial efficiency and a reduced oxygen cost during submaximal exercise, contributing to greater exercise economy and endurance performance [7,15,19,20]. Additionally, beetroot supplementation has been associated with improved cardiovascular responses and exercise efficiency, supporting athletic performance across a range of exercise modalities [1,14,21]. At the muscular level, nitrate supplementation supports energy production and metabolic efficiency, potentially facilitating training adaptations and recovery by reducing muscle fatigue and soreness [7,10,19,22,23]. However, the ergogenic response to beetroot supplementation is highly variable and influenced by factors such as training status, sex, and dosage, with evidence suggesting diminished effects in highly trained athletes [2,22,24]. Furthermore, although generally considered safe, excessive nitrate intake may raise concerns regarding the formation of potentially harmful compounds, highlighting the need for appropriate dosing strategies [15,23].

Despite growing research interest in beetroot-derived nitrate supplementation among elite athletes, several methodological limitations hinder definitive conclusions about its effects and population-level implications. Although existing studies have investigated beetroot-derived nitrate supplementation, highly trained and elite athletes remain underrepresented, and the optimal dose, timing, supplementation duration, and long-term effects have yet to be established. In this review, athlete classification was based on the Participant Classification Framework proposed by McKay et al., which classifies athletes according to their training status, competitive level, and sporting achievement [25]. Within this framework, highly trained athletes (Tier 3) typically engage in structured, high-volume training, possess substantial sport-specific experience, and compete at the national level. In contrast, elite athletes (Tier 4) are characterized by a higher standard of performance, generally involving international-level competition alongside near-maximal training and advanced sport-specific proficiency [25]. Distinguishing between these populations is important because athletes competing at higher performance tiers typically operate close to their physiological limits, where even small performance improvements may influence competitive success, qualification, or medal outcomes [25,26,27,28,29]. Moreover, repeated exposure to intensive training induces the repeated bout effect, a protective adaptation characterized by reduced exercise-induced muscle damage, inflammation, and perceived soreness following subsequent exercise bouts [30,31,32,33]. These long-term training adaptations may alter recovery kinetics and modify physiological responses to dietary nitrate supplementation, particularly those related to muscle function and recovery [34,35]. As a result, the efficacy of beetroot-derived nitrate in enhancing performance, physiological outcomes, and recovery may differ substantially between elite athletes and less-trained populations [7,8,16]. Given these unique physiological and performance characteristics, a comprehensive synthesis of randomized controlled trials conducted specifically in highly trained and elite athletes is warranted.

Therefore, the present scoping review aims to systematically map and synthesize the available evidence on beetroot-derived nitrate supplementation in highly trained and elite athletes, with particular emphasis on performance, recovery, and physiological outcomes.

## 2. Methods

This scoping review protocol was developed in accordance with the methodological guidance provided by the Joanna Briggs Institute for scoping reviews [36,37] and is reported following the Preferred Reporting Items for Systematic Reviews and Meta-Analyses extension for Scoping Reviews (PRISMA-ScR) (Supplementary File S1) [38]. This scoping review protocol was preregistered on the Open Science Framework (OSF) under embargo to maintain blinding during peer review (https://osf.io/2xjp7, accessed on 10 April 2026). The review aims to systematically map and synthesize evidence on the effects of beetroot-derived nitrate supplementation in highly trained and elite athletes.

### 2.1. Eligibility Criteria

Eligibility criteria were defined using the Population–Concept–Context (PCC) framework (Table 1), in accordance with the Joanna Briggs Institute’s guidance. Studies were considered eligible when they included athletes classified from highly trained to world-class (McKay Tier 3–5) [25]. The included studies examined the effects of beetroot-derived nitrate supplementation on performance, recovery, and physiological or biomarker responses within sport, exercise, or related settings. In addition, only randomized controlled trials (RCTs) were considered eligible. Detailed inclusion and exclusion criteria are presented in Table 1.

### 2.2. Information Sources and Search Strategy

A comprehensive literature search was conducted across multiple electronic databases, including Scopus, Web of Science, and PubMed. Initial database searches were conducted in March 2026, with an updated search completed on 14 May 2026. The search strategy combined keywords and Boolean operators related to beetroot-derived nitrate supplementation and athletic performance. A representative search string was: (“beetroot*” OR “beta vulgaris”) AND (athlete* OR sport* OR player* OR exercise OR “physical activity”) AND (“randomized controlled trial*” OR “randomised controlled trial*” OR “controlled clinical trial*” OR randomized* OR randomised OR RCT). The search strategy was adapted for each database according to its indexing terms and search requirements (Supplementary File S2). In addition, the reference lists of included studies were screened to identify any further relevant articles. Grey literature was not included to ensure the inclusion of peer-reviewed, methodologically robust randomized controlled trials. Restricting the search to peer-reviewed sources also enhanced transparency and reproducibility.

### 2.3. Study Selection

**Figure 1:**
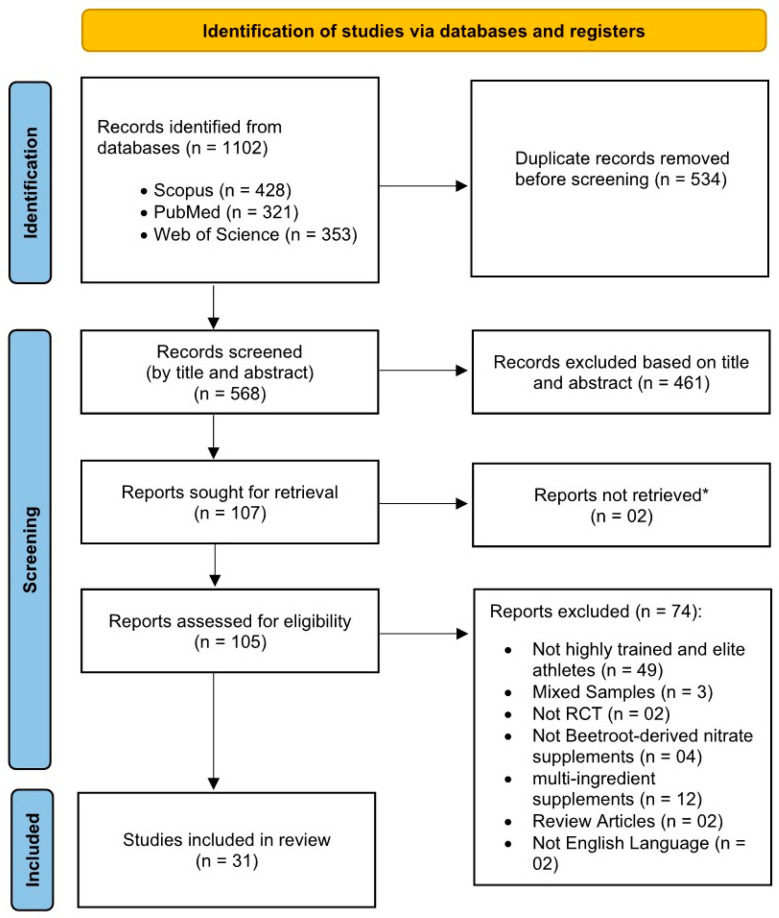
PRISMA flow diagram illustrating the study-selection process. * Two reports could not be retrieved despite comprehensive efforts including institutional subscriptions, author correspondence (*n* = 2, no responses), and searches across databases/platforms.

All identified records were imported into Microsoft Excel version 365 (Microsoft Corporation, Redmond, WA, USA), and duplicate records were removed before screening. Study selection was conducted in two stages. First, titles and abstracts were independently screened by two reviewers (N.C. and M.N.) against the predefined eligibility criteria. Second, full-text articles of potentially relevant studies were retrieved and assessed for inclusion. Discrepancies between reviewers were resolved through discussion, and a third reviewer (H.I.C.) was consulted where necessary. The study-selection process was documented using a PRISMA flow diagram (Figure 1).

### 2.4. Data Extraction and Charting

Data were extracted using a standardized data extraction form. The extracted information included study characteristics (author and year), objective, participant details (sport, sample size, competitive level), study design, supplementation protocol (dose, duration, and form), outcome measures (performance, recovery, and physiological variables), and key findings. Data extraction was performed independently by two reviewers to ensure accuracy and consistency.

## 3. Results and Discussion

### 3.1. Search Results

The database search yielded a total of 1102 records, comprising Scopus (*n* = 428), PubMed (*n* = 321), and Web of Science (*n* = 353). After removing 534 duplicate records, 568 unique records remained for title and abstract screening. During the initial screening phase, 461 records were excluded based on title and abstract, primarily due to irrelevance to the study objectives. This resulted in 107 reports being sought for full-text retrieval, of which 105 reports were successfully retrieved. In comparison, 2 reports could not be accessed despite extensive efforts, including searches of institutional databases and attempts to contact the corresponding authors. Full-text assessment led to the exclusion of 74 studies for the following reasons: participants not classified as highly trained or elite (*n* = 49), mixed samples without extractable data (*n* = 3), non-randomized study designs (*n* = 2), interventions not involving beetroot-derived nitrate (*n* = 4), use of multi-ingredient supplements (*n* = 12), review articles (*n* = 2), and non-English publications (*n* = 2). In the end, 31 studies met the eligibility criteria and were included in the final synthesis of this scoping review (Figure 1).

### 3.2. Study Characteristics

A total of 31 peer-reviewed articles were included. All the included studies performed beetroot-derived nitrate supplementation in highly trained and elite athletes. Most studies employed rigorous experimental methodologies, predominantly randomized, double-blind designs. In addition, a limited number of studies employed methodological approaches such as placebo-controlled cross-over trials and counterbalanced cross-over studies. These designs were reported in various sports, including water polo, field hockey, football, cycling, swimming, rowing, rugby, tennis, and athletics, demonstrating a strong attempt to mitigate bias and inter-individual variability. The cross-over design is appropriate given the limited number of participants and their highly trained nature.

**Figure 2:**
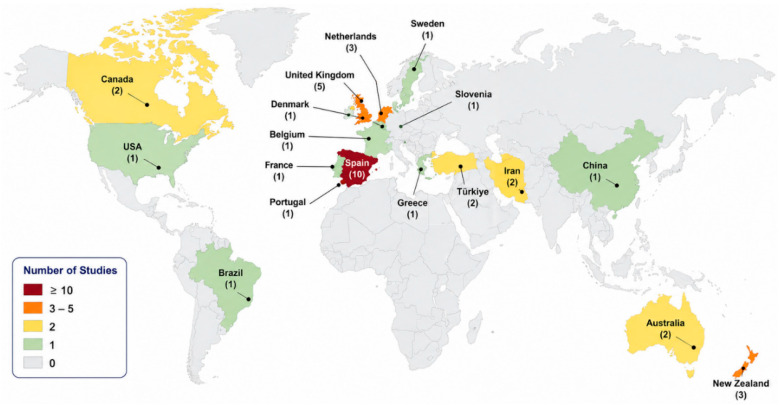
Geographical distribution of the included studies (*n* = 31). Note: countries involved in multiple studies across collaborations are counted individually. Source: prepared by the authors based on data extracted from the included studies.

The studies were conducted worldwide, primarily in countries such as Spain, the United Kingdom, the Netherlands, and New Zealand, demonstrating global interest in nitrate supplementation to enhance the performance of highly trained and elite athletes. The geographical distribution of the included studies is presented in Figure 2. The sample size spectrum ranges from 8 to 80 participants, and most studies included fewer than 20 participants. Thus, a lower number of participants might be due to difficulty recruiting elite-level athletes for the study. Among the selected studies, male participants were predominant, accounting for 23 studies, while 3 studies included female participants, particularly in water polo, field hockey, and rugby [39,40,41]. Studies conducted in swimming, winter triathlon, short-track speed skating, and cross-country skiing included participants of both genders. Most participants were young adults with a homogeneous age distribution ranging from 15 to 40 years (Table 2). Most studies focused on analyzing the acute effects of beetroot juice supplementation on exercise performance, physiological responses, neuromuscular function, recovery, and perceptual outcomes in elite athletes.

### 3.3. Training Characteristics

The included studies represented a diverse range of sports, including endurance sports (cycling, athletics, rowing, triathlon, and cross-country skiing), intermittent team sports (football (soccer), rugby, handball, field hockey, and water polo), racquet sports (tennis), combat sports (taekwondo), aquatic sports (swimming), and winter sports (short-track speed skating, winter triathlon and alpine slalom skiing) (Table 2). Endurance sports such as cycling, rowing, athletics, triathlon, and cross-country skiing were the most extensively investigated across the included studies. Among these, cycling emerged as the predominant sports discipline [3,42,43,44,45,46], accounting for six studies, thereby highlighting the considerable research interest in the effects of beetroot-derived nitrate supplementation on cycling performance and associated physiological outcomes. Several studies have also reported the effects of beetroot supplementation on athletes participating in intermittent team sports such as football (soccer) [47,48,49], rugby [40,50], handball [51], field hockey [41], and water polo [39].

Among the selected studies, substantial deviations were observed in supplementation, dose, timing, and duration. Most studies examined supplementation with concentrated beetroot, while a few used large volumes of beetroot juice. The nitrate dose ranged from 4 to 19.5 mmol (248–1209 mg) NO_3_^−^ per day. At the same time, many studies used a dose range of 6.4–12.8 mmol (397–794 mg) NO_3_^−^. Acute supplementation protocols were most common, with beetroot juice ingested 2–3 h before exercise testing, coinciding with the peak elevation in plasma nitrite concentration and nitric oxide bioavailability. Several investigations also implemented short-term loading strategies lasting 3–15 days, often involving daily supplementation to maintain elevated nitrate stores.

**Table 2:** Descriptive and intervention characteristics.

Art. No.	Authors & Year	Sport Type	Objective	Study Design	Sample Size (Sex, Age)	Form, Dose, Timing, and Duration	Placebo Composition	Outcomes Measured	Key Findings
1	Arnaoutis et al. 2026 [52]	Swimming	Effects of acute BRJ supplementation on swimming performance, blood lactate, and perceived exertion.	Randomized, double-blind, placebo-controlled, cross-over trial	*n* = 12 (male, 20.2 ± 2.2 years)	140 mL concentrated BRJ (Beet It Sport), ~8 mmol NO_3_^−^ (~496 mg), single acute dose 2 h before first 200-m trial; two 200-m TTs separated by 60 min	Custom NO_3_^−^ depleted drink matched for taste, color, and nutrients; included salt, fructose, organic pea protein, lemon juice, and food coloring; energy 114 kcal/140 mL, carbs 22.4 g, protein 6.0 g, salt 0.62 g	200-m front-crawl completion time (two trials), blood lactate, RPE, serum TNO_x_	No significant differences in 200-m times between BRJ and placebo in either trial or in change between trials (*p* > 0.05). No differences in lactate or RPE between conditions (*p* > 0.05). BRJ markedly increased serum TNO_x_ (~>10-fold vs. placebo) but without ergogenic effect.
2	Eroglu et al. 2026 [49]	Football (soccer)	Effects of acute BRJ supplementation on anaerobic power and vastus lateralis muscle oxygenation during the Wingate test.	Randomized, double-blind, placebo-controlled, cross-over trial	*n* = 16 (male, 18.2 ± 0.4 years)	140 mL concentrated BRJ (Beet-It Pro Elite Shot); contains 12.8 mmol (794 mg) NO_3_^−^; ingested 2.5 h before Wingate test; single acute dose	Low-calorie blackcurrant juice with negligible NO_3_^−^ (<0.1 mmol; <6.2 mg), same volume, in identical opaque bottles	Wingate peak power, mean power, time to peak power, fatigue index; muscle oxygenation (SmO_2_, HHb, tHb); blood lactate, glucose, blood pressure, HR, RPE	Peak power (~11%) and mean power (~7%) increased, while time to peak power (~10%) decreased. No effects on fatigue index, HR, blood pressure, or RPE. Exercise SmO_2_ was unchanged, but post-exercise SmO_2_ increased (~10%), HHb decreased (~13%), and blood lactate increased following BRJ supplementation.
3	Ahmadpour et al. 2024 [53]	Alpine slalom skiing	Effects of acute BRJ supplementation on slalom performance and muscle soreness.	Randomized, double-blind, placebo-controlled cross-over trial	*n* = 10 (male, 22 ± 3.6 years)	BRJ, 220 mL containing ~8.9 mmol (~552 mg) NO_3_^−^; ingested 2.5 h before testing (acute)	220 mL matched drink, <0.5 mmol (<31 mg) NO_3_^−^, similar color/taste with cherry coloring and sugar	90-s box jump (anaerobic capacity), hexagonal obstacle jump (agility), wall-sit (isometric endurance), total time of 2 runs at ~3000 m, RPE after each run, muscle soreness (visual analog scale) at baseline, 12, 24, 48 h post-runs	Box jumps (+7.6%), wall-sit time (+27.9%), and agility performance (+4.8%) improved. Slalom performance improved by 1.74%, but the improvement was not statistically significant. RPE decreased (−10.8%), and muscle soreness was reduced by 22–38% at 12, 24, and 48 h.
4	Garnacho-Castaño et al. 2024 [54]	Rowing	Effects of acute BRJ supplementation on rowing performance, cardiorespiratory, and metabolic responses.	Randomized, double-blind, placebo-controlled cross-over trial	*n* = 10 (male, 36.60 ± 4.97 years)	BRJ, Beet-It-Pro Elite Shot; 140 mL containing ~12.8 mmol NO_3_^−^ (~794 mg); single dose, 3 h before test; acute (one intake per trial day)	140 mL of a drink prepared from 2 g powdered BRJ in 1 L water plus lemon juice to mimic flavor/texture; very low NO_3_^−^ content	Performance: 2000-m time trial, mean power, strokes/min, meters per stroke. Cardiorespiratory: $\dot{V}O_{2}\max$ (absolute, relative), mean $\dot{V}O_{2}$, HR, VE, VE·VCO_2_−1 slope. Metabolic and perceptual: blood lactate, oxygen saturation, RPE.	Time-trial performance improved (~4 s), with increased absolute (+0.16 L·min^−1^) and relative (+2.10 mL·kg^−1^·min^−1^) $\dot{V}O_{2}\max$. No effects on ventilatory efficiency ($\dot{V}E$/$\dot{V}{\mathrm{CO}}_{2}$ slope), blood lactate, oxygen saturation, or RPE.
5	Moreno-Heredero et al. 2024 [55]	Swimming	Effects of acute BRJ supplementation on swimming performance, anaerobic responses, and perceptual measures during high-intensity interval exercise.	Randomized, cross-over, placebo-controlled, double-blind, counterbalanced trial	*n* = 18 (9 males and 9 females), 17.1 ± 1.2 years)	Beet-It-Pro Elite Shot BRJ; 70 mL, 6.4 mmol NO_3_^−^ (397 mg); single dose, 3 h pre-test; acute (one day, one ingestion)	70 mL, 0.04 mmol NO_3_^−^ (2.5 mg); matched flavor, dose, appearance, packaging	100 m split and total times over 2 × 6 × 100 m maximal front-crawl with 40 s intra-rep rest, 3 min between blocks; blood lactate (multiple time points), HR; CMJ pre/post; RPE, TQR	No significant differences between BRJ and placebo in partial or total times across the 2 × 6 × 100 m test. The BRJ swimmers improved their time on the 6th repetition in block 2 compared with block 1, suggesting greater tolerance at the very end. No between-condition differences in lactate or HR. RPE and TQR did not differ across conditions, but the BRJ group showed better perceived recovery at the end of the interval.
6	Muñoz et al. 2024 [51]	Handball	Effects of 3-day BRJ supplementation on neuromuscular performance.	Randomized, double-blind, placebo-controlled, cross-over trial	*n* = 12 (male, 21.5 ± 5.7 years)	BRJ shot (Beet-It-Pro Elite Shot); 70 mL/day providing 6.4 mmol NO_3_^−^ (397 mg); from beetroot (98%) and lemon juice (2%); taken once daily at 17:00 for 3 consecutive days, including test day	70 mL NO_3_^−^ depleted BRJ shot with identical composition, flavor, appearance, and packaging; 0.04 mmol (2.5 mg) NO_3_^−^	Salivary NO_3_^−^ and NO_2_^−^; neuromuscular tests: handball throwing velocity (7 m, 9 m), isometric handgrip strength, CMJ height, Modified Agility T-test time, repeated-sprint test (best time, mean time, sprint decrement); RPE; side effects questionnaire	BRJ significantly increased salivary NO_3_^−^ and NO_2_^−^; improved isometric handgrip strength by 7.8% and CMJ height by 4.7% vs. placebo; no significant effects on throwing velocity, agility T-test, or repeated-sprint indices; no RPE differences; only side effect increased was red urine prevalence.
7	Esen et al. 2023 [50]	Rugby	Effects of acute BRJ supplementation on modified Yo-Yo IR1 performance and countermovement jump performance.	Randomized, counterbalanced, double-blind, placebo-controlled cross-over design	*n* = 12, (male, 20 ± 4 years)	Concentrated BRJ (Beet It); 2 × 70 mL, ~12.8 mmol (~794 mg) NO_3_^−^ total; consumed 3 h before testing; single acute dose (one test day per condition)	Same product, NO_3_^−^ depleted BRJ (~0.08 mmol NO_3_^−^; ~5.0 mg NO_3_^−^)	Prone Yo-Yo IR1 total distance (intermittent running performance). CMJ height pre- and post-Yo-Yo IR1. Plasma nitrate and nitrite, blood lactate.	Plasma NO_3_^−^ and NO_2_^−^ increased markedly with BRJ vs. placebo. No significant difference in prone Yo-Yo IR1 distance between BRJ and placebo. CMJ height decreased after the test in both conditions, with no effect of the supplement.
8	Huang et al. 2023 [56]	Winter triathlon	Effects of 7-day high-nitrate BRJ supplementation on endurance performance.	Randomized, double-blind, placebo-controlled trial	*n* = 80 (44 males and 36 females), 21.12 ± 1.33 years)	Concentrated BRJ (“Beet It Sport”); 6.4 mmol (397 mg) NO_3_^−^/70 mL, three times per day (total 19.5 mmol (1209 mg) NO_3_^−^/day; 210 mL) for 7 days; timing for testing: last dose ~2 h before lab tests on day 8	NO_3_^−^ free BRJ, 0.065 mmol (4.0 mg) NO_3_^−^/70 mL, same brand/packaging, visually and organoleptically indistinguishable, no additional food components	Submaximal treadmill running (VO_2_, RER, HR, blood lactate, RPE at 60/70/80% VO_2_peak); cycling time-to-exhaustion at 85% peak power; 10-km cross-country skiing time trial	At the highest speed (V3), BRJ reduced VO_2_, RER, and blood lactate vs. placebo; no differences at lower speeds; HR and RPE were not significantly changed. BRJ significantly increased time to exhaustion in both males and females. No significant improvement in skiing performance.
9	Macuh et al. 2023 [48]	Football (soccer)	Effects of nitrate supplementation on performance and perceived exertion according to habitual dietary nitrate intake.	Randomized, placebo-controlled, double-blind, cross-over trial	*n* =15 (male, 23.3 ± 2.7 years)	70 mL concentrated BRJ, 6.4 mmol (397 mg) NO_3_^−^, single dose 2 h before Cooper test; each player did both nitrate and placebo conditions separated by a 2-week washout	70 mL BRJ drink, visually and taste matched, with NO_3_^−^ removed; “Beet It, Placebo Shots”	Distance covered in 12-min Cooper test (treadmill) and estimated $\dot{V}O_{2}\max$. RPE (Borg 6–20 scale) at 3, 6, 9, 12 min; dietary data: 3-day food diaries to quantify nitrate, energy, and macronutrient intake; mean NO_3_^−^ intake 2.66 ± 1.21 mmol (165 ± 75 mg) NO_3_^−^/day	Cooper test distance increased by ~5% (+0.145 km) in athletes with habitual NO_3_^−^ intake <4.84 mmol (<301 mg) NO_3_^−^/day. No effect on RPE. Insufficient data to evaluate athletes with habitual NO_3_^−^ intake >4.84 mmol (>301 mg) NO_3_^−^/day.
10	Moreno et al. 2023 [57]	Swimming	Effects of acute BRJ supplementation on repeated maximal swimming performance.	Randomized, placebo-controlled, double-blind, cross-over trial	*n* = 13 (7 males, 6 females, 15.8 ± 1.7 years)	70 mL Beet-It Pro Elite BRJ shot, 6.4 mmol (397 mg) NO_3_^−^; ingested once, 3 h before test; acute (single dose)	70 mL NO_3_^−^ depleted BRJ, 0.04 mmol (2.5 mg) NO_3_^−^, matched in flavor, appearance, and packaging	50 m and 100 m times; blood lactate; RPE; TQR; HR; CMJ; underwater distance and velocity; stroke frequency	No significant overall performance differences in 6 × 100 m times, lactate, HR, RPE, or CMJ between BRJ and placebo; possible small improvement in last 100 m time and in early-repetition RPE/recovery; slightly greater start underwater distance with beetroot in some repetitions.
11	Fernández-Elías et al. 2022 [58]	Tennis	Effects of acute BRJ supplementation on movement patterns during competitive tennis match play.	Randomized, double-blind, placebo-controlled cross-over trial	*n* = 9 (male, 24.9 ± 4.2 years)	70 mL concentrated BRJ shot (Beet IT) containing 6.4 mmol (397 mg) NO_3_^−^, ingested once, 3 h before a 3-set tennis match; acute, single-day protocol	70 mL drink with 1 g powdered BRJ and lemon juice in water, similar taste/color, ~0.005 mmol (~0.31 mg) NO_3_^−^	Match-play running demands via GPS (speed zones, total distance, accelerations/decelerations, impacts, peak velocity); serve speed; isometric handgrip strength; RPE	BRJ did not change any match-play running variable, serve speed, handgrip strength, or RPE compared with placebo; concluded that acute NO_3_^−^ rich BRJ has little value for enhancing physical performance in professional tennis players.
12	López-Samanes et al. 2022 [40]	Rugby	Effects of acute BRJ supplementation on neuromuscular performance.	Randomized, double-blind, placebo-controlled cross-over trial	*n* = 14 (female, 25.0 ± 3.7 years)	BRJ (Beet-It-Pro Elite Shot), 140 mL containing 12.8 mmol (794 mg) NO_3_^−^, ingested 2.5 h before testing; acute, single-dose per trial	140 mL BRJ-based placebo, matched for flavor, appearance, packaging, containing 0.08 mmol (5.0 mg) NO_3_^−^	CMJ; dominant isometric handgrip strength; 10 m and 30 m sprint; modified agility *t*-test; Bronco (1.2 km shuttle) endurance test; RPE (muscular, cardio, general); side-effects questionnaire	BRJ significantly improved CMJ height. No significant effects on handgrip strength, 10 m or 30 m sprint, agility *t*-test, Bronco endurance test, or any RPE measures. Higher prevalence of gastrointestinal upset with BRJ.
13	López-Samanes et al. 2022 [41]	Field hockey	Effects of acute BRJ supplementation on neuromuscular performance and match-play demands.	Randomized, double-blind, placebo-controlled cross-over trial	*n* = 11 (female, 22.8 ± 5.1 years)	BRJ, 70 mL, 6.4 mmol (397 mg) NO_3_^−^ (Beet-It-Pro Elite Shot); single acute dose, ingested 3 h before testing on each of two separate days, one week apart	70 mL NO_3_^−^ depleted BRJ (0.04 mmol NO_3_^−^; 2.5 mg NO_3_^−^), matched in flavor, appearance, and packaging	Neuromuscular tests: CMJ, isometric handgrip strength, 20 m sprint, repeated sprint ability; match-play: GPS-derived distances at different speeds, peak velocity, number of high-intensity accelerations/decelerations; session RPE	Acute 70 mL BRJ (6.4 mmol NO_3_^−^; 397 mg NO_3_^−^) did not produce statistically significant improvements in neuromuscular performance, GPS match-play variables, or perceived exertion compared with placebo in elite female field hockey players.
14	Dumar et al. 2021 [59]	Athletics	Effects of acute BRJ supplementation on morning-associated declines in anaerobic performance during Wingate sprints.	Double-blind, randomized, placebo-controlled, counterbalanced cross-over trial	*n* = 10 (male, 20.3 ± 1.9 years)	70 mL concentrated BRJ “Sport shot, Beet It”; ~6.4 mmol (~397 mg) NO_3_^−^; 2 h pre-exercise, consumed within 5 min; single acute dose per AM trial	70 mL blackcurrant juice (negligible NO_3_^−^)	Mean power, anaerobic capacity, total work (3 × 15 s Wingate tests), HR, RPE	Morning placebo showed significantly lower mean power, anaerobic capacity, and total work than the afternoon placebo. With BRJ in the morning, these decrements were abolished; performance was similar to that of the afternoon. HR was lower in AM-BRJ than in AM-placebo and PM, while RPE did not differ across conditions.
15	Miraftabi et al. 2021 [60]	Taekwondo	Effects of acute nitrate-rich BRJ supplementation on taekwondo-specific performance, physiological responses, and cognitive function.	Randomized, double-blind, placebo-controlled, cross-over	*n* = 8 (male, 20 ± 4 years)	BRJ “Red Beet Vinitrox Shot”, 60 mL bottles, 6.4 mmol (397 mg) NO_3_^−^ per bottle; BJ-400: 60 mL BJ + 60 mL placebo (6.4 mmol NO_3_^−^; 397 mg NO_3_^−^ total); BJ-800: 120 mL BJ (two bottles, 12.9 mmol (800 mg) NO_3_^−^ total) Ingested 2.5 h before tests; acute (single-day) dosing	NO_3_^−^ depleted beetroot powder drink (1 g dried beetroot powder per liter water + lemon juice), identical bottles	Aerobic: Progressive Specific Taekwondo Test (time to exhaustion); anaerobic: Multiple Frequency Speed of Kick Test, CMJ (height, power, etc.); physiological: blood lactate, HR, RPE; cognitive: Stroop word–color test	No significant differences between BJ-400 (6.4 mmol NO_3_^−^; 397 mg NO_3_^−^), BJ-800 (12.9 mmol NO_3_^−^; 800 mg NO_3_^−^), placebo, and control for taekwondo-specific aerobic or anaerobic performance, CMJ, blood lactate, HR, or RPE. Cognitive function after PSTT was higher in the BJ-400 (6.4 mmol NO_3_^−^; 397 mg NO_3_^−^) treatment than in the other treatments. More gastrointestinal symptoms, especially with BJ-800 (12.9 mmol NO_3_^−^; 800 mg NO_3_^−^); none in placebo/control.
16	López-Samanes et al. 2020 [61]	Tennis	Effects of BRJ supplementation on tennis-specific physical performance.	Randomized, double-blind, placebo-controlled, cross-over trial	*n* = 13 (male, 25.4 ± 5.1 years)	BRJ shot (Beet-It-Pro Elite Shot, 70 mL), 6.4 mmol (397 mg) NO_3_^−^ per 70 mL, single dose, 3 h before neuromuscular test battery, acute, one ingestion per trial; two test days (BRJ vs. placebo) separated by 1 week	70 mL low NO_3_^−^ drink (0.04 mmol NO_3_^−^; 2.5 mg NO_3_^−^; ECO Saludviva, Spain)	SVT, CMJ, IHS, 5-0-5 agility (dominant and non-dominant), 10-m sprint; RPE and environmental conditions	70 mL BRJ did not significantly improve any performance variable versus placebo (serve velocity, jump height, handgrip strength, agility, sprint); low-dose NO_3_^−^ did not enhance tennis physical performance in these players
17	Pawlak-Chaouch et al. 2019 [62]	Athletics and triathlon	Effects of BRJ supplementation on tolerance to supramaximal intermittent exercise.	Randomized, single-blind, placebo-controlled, cross-over trial	*n* = 9 (male, 21.7 ± 3.7 years)	BRJ, ~10.9 mmol (~676 mg) NO_3_^−^/L; 500 mL morning on 2 days prior + 500 mL 2 h pre-test (~5.2 mmol NO_3_^−^; ~322 mg NO_3_^−^/day for 3 days)	Apple–blackcurrant juice, NO_3_^−^ < 0.08 mmol/L (<5.0 mg NO_3_^−^/L); slightly higher energy (225 vs. 185 kcal)	Performance: number of 15-s cycling bouts at 170% maximal aerobic power until exhaustion; total work, mean power. Physiology: VO_2_, VCO_2_, ventilation, HR; muscle oxygenation (HbO_2_, HHb, tHb via NIRS); plasma NO_x_; blood pressure.	Plasma NO_x_ levels were much higher with BRJ than with placebo, confirming effective NO_3_^−^ loading. No performance benefit: repetitions completed and total work were identical between conditions. No physiological changes: VO_2_, ventilation, HR, and NIRS-derived muscle oxygenation indices did not differ between BRJ and placebo.
18	Rokkedal-Lausch et al. 2019 [3]	Cycling	Effects of 7-day high-dose BRJ supplementation on 10-km cycling time-trial performance and physiological responses under normoxic and hypoxic conditions.	Randomized, double-blinded, counterbalanced cross-over trial	*n* = 12 (male, 29.1 ± 7.7 years)	BRJ concentrate (Beet It Sport), 140 mL/day ~12.4 mmol (~769 mg) NO_3_^−^, split 70 mL morning + 70 mL evening for 7 consecutive days; on TT days, full 140 mL consumed ~2 h before lab (~2.75 h before TT)	140 mL/day NO_3_^−^ depleted BRJ (~0 mmol (0 mg) NO_3_^−^; from the same manufacturer	10-km TT power output and completion time, plasma NO_3_^−^/NO_2_^−^, VO_2_, % $\dot{V}O_{2}\max$, VCO_2_, VE, HR, SpO_2_, muscle oxygenation (NIRS variables), exercise efficiency (PO/VO_2_)	BRJ significantly increased plasma NO_3_^−^/NO_2_^−^ and improved TT performance: ~1.6% higher power and 0.6% faster time overall, with similar effects in normoxia and hypoxia; VO_2_ and VE during TT were higher, but exercise efficiency (PO/VO_2_), HR, SpO_2_, and muscle oxygenation were unchanged
19	Balsalobre-Fernández et al. 2018 [63]	Athletics	Effects of 15-day nitrate-rich BRJ supplementation on physiological, psychological, and biomechanical responses.	Double-blind, randomized, placebo-controlled trial	*n* = 12 (male, 26.3 ± 5.1 years)	BRJ shots, high-NO_3_^−^ 6.5 mmol (403 mg) NO_3_^−^ per 70 mL; 1 shot daily with breakfast for 15 consecutive days.	NO_3_^−^ depleted BRJ from the same manufacturer, 0.065 mmol (4.0 mg) NO_3_^−^/70 mL, identical packaging, color, smell, taste	Running economy (VO_2_ at 15, 17.1, 20 km/h), $\dot{V}O_{2}\max$, heart rate, respiratory exchange ratio, time to exhaustion, vastus lateralis muscle oxygen saturation (SmO_2_), leg stiffness, RPE (1–10 scale)	RPE, SmO_2_, and time to exhaustion improved. No effects on running economy, $\dot{V}O_{2}\max$, HR, or leg stiffness.
20	Garnacho-Castaño et al. 2018 [64]	Triathlon	Effects of acute BRJ supplementation on endurance performance and cardiorespiratory responses.	Randomized, double-blind, placebo-controlled trial, cross-over design	*n* = 12 (male, 39.3 ± 7.5 years)	BRJ concentrate (Beet-It-Pro Elite Shot), 70 mL (~6.5 mmol NO_3_^−^; 403 mg NO_3_^−^), single acute dose, ingested 3 h before testing	NO_3_^−^ depleted BRJ: 1 g powdered beetroot (~0.005 mmol NO_3_^−^); ~0.31 mg NO_3_^−^) in 1 L mineral water plus lemon juice	Cardioventilatory variables (VO_2_, VCO_2_, VE, HR, ventilatory equivalents), VO_2_ kinetics (slow component), cycling efficiency and gross mechanical efficiency, VT2 time-trial performance, blood lactate, energy expenditure, carbohydrate and fat oxidation, RPE	No significant effects of acute BRJ on cardioventilatory variables, efficiency/economy, VO_2_ slow component, VT2 time trial, energy expenditure, substrate oxidation, lactate, or RPE; authors suggest higher doses or longer supplementation may be needed in well-trained triathletes.
21	Jonvik et al. 2018 [39]	Water polo	Effects of dietary nitrate-rich BRJ supplementation on dynamic apnea and intermittent sprint performance.	Randomized, double-blind, cross-over trial	*n* = 14 (female; 22 ± 4 years)	NO_3_^−^ rich BRJ, 140 mL/day (12.9 mmol NO_3_^−^; 800 mg NO_3_^−^/day) for 6 days; 5 days at home + 6th dose on test day, tests ~3 h post-ingestion	NO_3_^−^ depleted BRJ (NO_3_^−^ depleted (PLA) BRJ)	Dynamic apnea test (max distance front crawl without breathing; distance, time-to-exhaustion, speed); intermittent 4 × 4 × 15 m swim sprint test (total time)	BRJ markedly increased plasma and salivary NO_3_^−^/NO_2_^−^, but did not improve intermittent sprint performance; overall, there was no significant effect on dynamic apnea, though a possible small benefit when BRJ was taken in the second period (test–order interaction).
22	McQuillan et al. 2018 [42]	Cycling	Effects of 3-day nitrate supplementation on thermoregulation, perceptual responses, and cycling time-trial performance in the heat.	Randomized, double-blind, placebo-controlled cross-over trial	*n* = 8 (male, 25 ± 8 years)	NO_3_^−^ rich BRJ, 140 mL/day (~8.0 mmol NO_3_^−^; ~496 mg NO_3_^−^), given as 2 × 70 mL per day, for 3 days; juice consumed ~90 min before lab visits	NO_3_^−^ rich depleted (~0.003 mmol NO_3_^−^; ~0.19 mg NO_3_^−^), identical taste, smell, and appearance to active juice	Serum nitrite, rectal temperature, HR, sweat loss (body mass change), 4-km time-trial mean power and time, RPE, thermal comfort, thermal sensation, feeling	NO_3_^−^ increased serum NO_2_^−^ and caused small increases in rectal temperature during low–moderate intensity cycling but did not change 4-km time-trial performance, HR, sweat rate, or most perceptual measures in the heat.
23	Richard et al. 2018 [65]	Short-track speed skating	Effects of 5-day nitrate supplementation on repeated 1000-m time-trial performance and recovery.	Double-blind, randomized, placebo-controlled, cross-over trial	*n* = 9 (4 males, 5 females), 21.8 ± 2.4 years)	High dose—115 mL juice blend incl. Beet It Sport, ~6.5 mmol (403 mg) NO_3_^−^/day; double dose (230 mL) 2.75–2.25 h pre–trial 1, 5 days; low dose—115 mL juice blend incl. Love Beets, ~0.9 mmol (~56 mg) NO_3_^−^/day, same schedule, 5 days	LO condition used BRJ with much lower (0.89 mmol NO_3_^−^; 55 mg NO_3_^−^) NO_3_^−^ plus fruit juices with negligible nitrate (0.01–0.03 mmol NO_3_^−^); 0.62–1.86 mg NO_3_^−^) but similar flavoring to HI	1000-m time-trial times (two efforts) and lap times. Plasma lactate 3- and 22-min post-race. HR (mean and peak) during trials. Salivary nitrate and nitrite before each trial.	Salivary NO_3_^−^/NO_2_^−^ increased by ~350–550% with high-NO_3_^−^ supplementation. No improvements in time-trial performance, plasma lactate, or HR.
24	McQuillan et al. 2017 [43]	Cycling	Effects of 6–8-day dietary nitrate (BRJ) supplementation on physiological responses and 4-km cycling time-trial performance.	Double-blind, placebo-controlled, cross-over trial	*n* = 8 (male, 26 ± 8 years)	70 mL NO_3_^−^ rich BRJ, ~4 mmol (~248 mg) NO_3_^−^ per 70 mL; one bottle daily for 8 days; on trial days, taken 2 h pre-test	NO_3_^−^ depleted BRJ (~0.003 mmol·L^−1^; ~0.19 mg NO_3_^−^·L^−1^), identical taste, smell, appearance, same manufacturer	4-km time-trial time, mean power output, HR, $\dot{V}O_{2}\mathrm{peak}$, incremental peak power output, ventilatory thresholds (VT1, VT2), cycling economy, serum NO_2_^−^	4-km time-trial performance (−0.7%) and mean power (+2.4%) improved, with no meaningful changes in $\dot{V}O_{2}\mathrm{peak}$, VT1, VT2, or cycling economy, suggesting performance benefits without clear physiological adaptations.
25	Nyakayiru et al. 2017 [47]	Soccer (football)	Effects of 6-day nitrate-rich BRJ supplementation on high-intensity intermittent exercise performance.	Randomized, double-blind, placebo-controlled, cross-over study	*n* = 32 (male, 23 ± 1 years)	BRJ shots, 2 × 70 mL/day (total 140 mL/day) NO_3_^−^ rich BRJ providing ~12.9 mmol (~800 mg) NO_3_^−^, for 6 days; last bolus 3 h before test, participants reported ~2 h after last bolus, tests ~2.5–3 h after ingestion	NO_3_^−^ depleted BRJ, identical in taste and appearance to an active drink	Primary: distance covered in Yo-Yo IR1; secondary: plasma and saliva NO_3_^−^/NO_2_^−^ concentrations, mean and peak HR, gastrointestinal tolerance, RPE	Plasma and salivary NO_3_^−^/NO_2_^−^ increased, Yo-Yo IR1 distance improved (+3.4%), and mean HR decreased. No effects on peak HR, RPE, or gastrointestinal tolerance.
26	Nybäck et al. 2017 [66]	Cross-country skiers (roller-skiing)	Effects of acute high-dose nitrate-rich BRJ supplementation on submaximal physiological responses and 1000-m time-trial performance under normoxic and hypoxic conditions.	Randomized, double-blind, cross-over trials	*n* = 8 (5 males and 3 females), 21.4 ± 2.4 years)	140 mL concentrated BRJ, ~13 mmol (~806 mg) NO_3_^−^, 2.5 h pre-exercise; acute, single dose	NO_3_^−^ depleted BRJ, ~0 mmol (0 mg) NO_3_^−^.	Plasma NO_3_^−^/NO_2_^−^, blood pressure, muscle oxygenation, VO_2_, HR, lactate, SpO_2_, RPE during two 6-min submaximal bouts and a 1000 m time trial.	BRJ increased plasma NO_3_^−^ and NO_2_^−^ levels but did not alter submaximal VO_2_, HR, lactate, RPE, muscle oxygenation, or blood pressure. The 1000 m time trial remained unchanged in both normoxia and hypoxia.
27	Boorsma et al. 2014 [67]	Athletics	Effects of acute and chronic BRJ supplementation on submaximal $\dot{V}O_{2}$ and 1500-m time-trial performance.	Randomized, double-blind, placebo-controlled cross-over trial	*n* =8 (male, 23.8 ± 5 years)	Concentrated BRJ (Beet It Sport); acute test days: 210 mL BRJ (19.5 mmol NO_3_^−^; 1209 mg NO_3_^−^) 2.5 h pre-TT; days 2–7: 140 mL BRJ (13.0 mmol NO_3_^−^; 806 mg NO_3_^−^) daily; total duration per phase 8 days	NO_3_^−^ free BRJ passed through ion-exchange resin; 0.065 mmol (4.0 mg) NO_3_^−^/70 mL; identical packaging, taste, smell, appearance	VO_2_ during submaximal treadmill running at 50, 65, 80% VO_2_peak; 1500 m time-trial time, plasma NO_3_^−^, VCO_2_, RER, HR, individual responder analysis, blinding	BRJ greatly increased plasma NO_3_^−^ but did not change VO_2_ at any running intensity and did not improve mean 1500 m performance vs. placebo. Two of 8 runners showed meaningful individual performance improvements and reduced VO_2_ (responders)
28	Hoon et al. 2014 [68]	Rowing	Effects of moderate- and high-dose nitrate-rich BRJ supplementation on 2000-m rowing ergometer performance.	Placebo-controlled, double-blind, randomized cross-over trail	*n* = 10 (male, 20.6 ± 2.5 years)	Commercial BRJ (Beet It); three conditions: 0 mmol (0 mg) NO_3_^−^ (placebo), 4.2 mmol (260 mg) NO_3_^−^ (SINGLE), 8.4 mmol (521 mg) NO_3_^−^ (DOUBLE) NO_3_^−^, achieved via two 70 mL bottles 2 h before the 2000-m test; acute, single-day dosing each trial	NO_3_^−^ depleted BRJ, identical in taste, appearance, and packaging	2000-m time, HR, post-exercise blood lactate, plasma NO_3_^−^/NO_2_^−^ before vs. 2 h after ingestion	A double dose of BRJ improved 2000-m time-trial performance, whereas a single dose had no meaningful effect. Plasma NO_3_^−^/NO_2_^−^ increased dose-dependently and was associated with greater performance improvement.
29	Hoon et al. 2014 [44]	Cycling	Effects of nitrate supplementation and dosing timing on repeated high-intensity cycling performance.	Placebo-controlled, randomized cross-over design	*n* = 26 (male, 20.3 ± 1.4 years)	NO_3_^−^ rich BRJ concentrate (Beet-it); 70 mL (4.1 mmol NO_3_^−^/254 mg NO_3_^−^) per serving; TOP-UP added 35 mL; Beverages at 150 and/or 75 min pre-TT1; extra half-dose 75 min pre-TT2, depending on condition (150-PRE, 75-PRE, TOP-UP)	NO_3_^−^ depleted BRJ, identical taste/texture/packaging, 0.03 mmol (1.9 mg) NO_3_^−^	4-min TT mean PO (W) in TT1 and TT2, plasma NO_2_^−^ concentration	Plasma NO_2_^−^ increased, peaking 75–150 min post-ingestion. No improvement in the first 4-km time trial; second time-trial performance was slightly impaired. Overall, nitrate supplementation showed no ergogenic benefit.
30	Cermak et al. 2012 [45]	Cycling and triathlon	Effects of a single bolus of concentrated nitrate-rich BRJ supplementation on 1-h cycling time-trial performance.	Double-blind, randomized, repeated-measures cross-over trial	*n* = 20 (male, 26 ± 1 years)	Concentrated BRJ; 140 mL; 8.72 mmol (541 mg) NO_3_^−^; Ingested with breakfast, 2.5 h before time trial; single dose (acute)	140 mL NO_3_^−^ depleted BRJ; negligible NO_3_^−^ (0.004 mmol; 0.25 mg); similar taste/appearance	~1-h cycling time-trial performance (time to complete fixed work ~1073 kJ), PO, HR, cadence, plasma NO_3_^−^/NO_2_^−^, glucose, insulin, lactate, free fatty acids	Plasma NO_3_^−^ and NO_2_^−^ were substantially higher after BRJ vs. placebo before exercise. No improvement in time-trial time, mean power, HR, or cadence between beetroot and placebo trials. No differences in metabolic markers and no relationship between nitrite change and performance change.
31	Lansley et al. 2011 [46]	Cycling	Effects of acute dietary nitrate-rich BRJ supplementation on plasma nitrite, physiological responses, and cycling time-trial performance.	Randomized, double-blind, placebo-controlled trial	*n* = 9 (male, 21 ± 4 years)	BRJ, 0.5 L containing ~6.2 mmol (~384 mg) NO_3_^−^; ingested over 15 min, ~2.5–2.75 h before TT; acute single dose	0.5 L NO_3_^−^ depleted BRJ (~0.0047 mmol NO_3_^−^; ~0.29 mg NO_3_^−^), matched for appearance, odor, taste, texture	Plasma NO_2_^−^, blood pressure, power output, VO_2_, PO/VO_2_ ratio, time to complete 4- and 16.1-km TTs, VO_2_peak	Plasma NO_2_^−^ increased (+138%), systolic blood pressure decreased, mean power output increased (5–6%), and 4-km and 16.1-km time-trial performance improved (2.8% and 2.7%, respectively). No change in $\dot{V}O_{2}$.

Note: BRJ = beetroot juice; mL = millilitres; kcal = kilocalories; mmol = millimoles; mg = milligram; TT = time trial; RPE = Rating of Perceived Exertion; TNO_x_ = total nitrate and nitrite; SmO_2_ = muscle oxygen saturation; HHb = deoxyhaemoglobin; tHb = total haemoglobin; NO_3_^−^ = nitrate; NO_2_^−^ = nitrite; $\dot{V}O_{2}$ = oxygen uptake; $\dot{V}O_{2}\max$ = maximal oxygen uptake; $\dot{V}E$ = minute ventilation; $\dot{V}{\mathrm{CO}}_{2}$ = carbon dioxide production; $\dot{V}E$/$\dot{V}{\mathrm{CO}}_{2}$ slope = ventilatory efficiency; kg^−1^ = per kilogram of body mass; min^−1^ = per minute; CMJ = Countermovement Jump; TQR = Total Quality Recovery; IR1 = Intermittent Recovery Test Level 1; RER = respiratory exchange ratio; HR = heart rate; $\dot{V}O_{2}\mathrm{peak}$ = peak oxygen uptake; GPS = Global Positioning System; AM trial = morning trial; SVT = Serve Velocity Test; IHS = Isometric Handgrip Strength; HbO_2_ = oxyhaemoglobin; NIRS = near-infrared spectroscopy; NO_x_ = nitric oxide metabolites (nitrate and nitrite); SpO_2_ = peripheral oxygen saturation; PO = power output; VT2 = second ventilatory threshold; PLA = placebo; LO = low nitrate; HI = high nitrate; VT1 = first ventilatory threshold; km = kilometre.

### 3.4. Performance Outcomes

#### 3.4.1. Endurance

Several studies have reported the impact of beetroot-derived nitrate supplementation on endurance performance in elite athletes. It was noted that beetroot-derived nitrate supplementation yields inconsistent findings across studies (Table 2). A total of five studies reported improvements in time-trial performance [3,43,46,54,68], two studies observed enhanced exercise tolerance [56,63], and one study reported improved exercise efficiency following beetroot-derived nitrate supplementation [46]. A significant improvement in 4-km and 16.1-km cycling time-trial performance, accompanied by increased power output and improved power-to-oxygen consumption ratio, followed by the consumption of 0.5 L of beetroot juice (6.2 mmol NO_3_^−^; 384 mg NO_3_^−^) [46]. Likewise, 7 days of beetroot-derived nitrate consumption (12.4 mmol NO_3_^−^·day^−1^; 769 mg NO_3_^−^·day^−1^) considerably enhanced 10-km cycling time-trial performance [3]. McQuillan et al. [43] also reported a beneficial effect on 4-km cycling time-trial performance following 8 days of supplementation with approximately 4 mmol NO_3_^−^·day^−1^; 248 mg NO_3_^−^·day^−1^). However, these findings were not consistent across all studies, as four investigations reported no significant ergogenic benefits on time-trial performance following beetroot-derived nitrate supplementation [44,45,52,67].

Positive findings were also observed in intermittent endurance protocols. Nyakayiru et al. [47] found that six days of nitrate-rich beetroot juice (140 mL; ~12.9 mmol NO_3_^−^·day^−1^; 800 mg NO_3_^−^·day^−1^) improved Yo-Yo IR1 performance and reduced mean heart rate, indicating enhanced cardiovascular efficiency during high-intensity intermittent exercise in trained soccer players. Huang et al. [56] found that, following chronic high-dose supplementation (6.5 mmol NO_3_^−^; 403 mg NO_3_^−^), there was no improvement in skiing time-trial performance.

Contrary to these findings, several studies reported that beetroot supplementation significantly increased plasma nitrate and/or nitrite concentrations without reflecting any considerable improvement in cycling or running performance [45,62,64,67]. This inconsistency in the impact of nitrate supplementation among elite athletes suggests reduced responsiveness, potentially due to their superior baseline aerobic efficiency, higher endogenous nitric oxide production, and well-developed oxidative metabolism. In addition, variations in exercise modalities, environmental conditions, and supplementation protocols may explain variability in endurance-related outcomes.

#### 3.4.2. Sprint and Anaerobic Performance

The beneficial effect of beetroot-derived nitrate supplementation on short-duration, high-intensity exercise performance was postulated by several authors (Table 2). Eroglu et al. [49] postulated that 140 mL of beetroot (12.8 mmol NO_3_^−^; 794 mg NO_3_^−^) consumption had a considerable positive effect on the peak and mean power output during Wingate testing, accompanied by faster time to peak power and improved post-exercise muscle oxygenation in football players. Likewise, 220 mL of beetroot juice (8.9 mmol NO_3_^−^; 552 mg NO_3_^−^) significantly enhanced anaerobic performance, including more box-jump repetitions, improved agility, and prolonged wall-sit endurance in alpine skiers at moderate to high altitudes [53]. Interestingly, Dumar et al. [59] observed that consuming 70 mL of concentrated beetroot juice substantially abolished morning-related performance decline, resulting in a higher power output comparable to afternoon performance. Similarly, in highly trained rowers, a higher dose of nitrate (8.4 mmol NO_3_^−^; 521 mg NO_3_^−^) potentially augmented 2000 m rowing performance, whereas a lower dose (4.2 mmol NO_3_^−^; 260 mg NO_3_^−^) did not affect any meaningful outcome [68].

While studies conducted in tennis players [58,61], swimmers [55,57], field hockey players [41], and taekwondo [60] athletes reported no significant improvements in sprint speed, agility, repeated sprint ability, or sport-specific anaerobic tasks following an acute dose of nitrate supplementation. The lack of significant performance improvement with beetroot supplementation may be associated with the relatively low nitrate dose used in the intervention [61]. Further, it indicates that nitrate supplementation provides greater benefits in moderately trained individuals or in exercise protocols involving repeated high-intensity efforts rather than in highly specialized, sport-specific tasks.

#### 3.4.3. Neuromuscular Performance

Selected studies showed that beetroot-derived nitrate supplementation had mixed effects on neuromuscular performance in elite athletes, depending on the dose and type of training (Table 2). A 12.8 mmol (794 mg) NO_3_^−^ consumption significantly augmented neuromuscular performance, such as countermovement jump height in female rugby players [40]. Likewise, 6.4 mmol (397 mg) NO_3_^−^ supplementation in male handball players resulted in a meaningful improvement in countermovement jump performance, followed by a 3-day supplementation period [51]. Further, Ahmadpour et al. [53] demonstrated improvements in agility and isometric muscular endurance, suggesting enhanced muscle contractile efficiency and fatigue resistance. These findings support the proposed role of nitric oxide in improving calcium handling within skeletal muscle and enhancing type II muscle fiber contractility [69].

In contrast, several studies failed to demonstrate significant improvements in neuromuscular outcomes, such as sprint speed, handgrip strength, agility, or jumping performance, following beetroot-derived nitrate supplementation. Studies conducted on highly competitive tennis athletes [61], female hockey players [41], etc., consistently showed a trivial or negligible impact of acute nitrate supplementation on neuromuscular performance. This discrepancy in the effect of beetroot-derived nitrate supplementation on the neuromuscular performance of elite athletes might be due to variation in dose, training status, exercise specificity, and individual responsiveness to supplementation.

#### 3.4.4. Oxygen Consumption

Beetroot-derived nitrate supplementation elicited a mixed response in oxygen consumption among highly trained elite athletes. Few studies have demonstrated reduced oxygen consumption during submaximal exercise and improvements in exercise efficiency. Huang et al. [56] observed lower VO_2_, respiratory exchange ratio, and blood lactate during high-speed running after chronic nitrate supplementation (6.5 mmol NO_3_^−^; 403 mg NO_3_^−^) in winter triathletes. Likewise, Lansley et al. [46] also found improved power output relative to oxygen consumption, indicating enhanced muscular efficiency. However, no significant enhancement in VO_2_ and exercise economy was observed in runners [66,67] and triathletes [64]. The lack of consistent improvements in elite athletes may result from their already highly efficient aerobic systems, leaving limited scope for nitrate-mediated enhancement.

#### 3.4.5. Recovery and Perceptual Outcomes

Recovery outcomes refer to indicators of post-exercise recovery, such as muscle soreness, muscle oxygenation, and perceived recovery, whereas perceptual outcomes include subjective measures such as ratings of perceived exertion and recovery status [70]. Only a few studies have investigated the effects of beetroot-derived nitrate supplementation on these outcomes in highly trained and elite athletes, and the findings remain inconsistent. Ahmadpour et al. [53] reported that acute ingestion of 220 mL of beetroot juice (8.9 mmol NO_3_^−^; 552 mg NO_3_^−^) significantly reduced muscle soreness and ratings of perceived exertion in alpine skiers. Similarly, Eroglu et al. [49] observed improved post-exercise muscle oxygenation in elite football players. In contrast, Moreno-Heredero et al. [55] reported no significant effects on recovery-related variables following acute ingestion of 70 mL of beetroot juice (6.4 mmol NO_3_^−^; 397 mg NO_3_^−^) in competitive swimmers.

### 3.5. Limitations and Future Directions

The findings of this scoping review should be interpreted in light of several limitations. Considerable heterogeneity was observed across the included studies in terms of sports disciplines, supplementation doses, formulations, timing, duration, and outcome measures, limiting direct comparisons. Furthermore, many studies included relatively small cohorts of highly trained and elite athletes, reflecting the practical challenges of recruiting these populations. Consequently, the findings should be interpreted with caution, as subtle performance effects may not have been consistently detected across studies. In addition, most studies enrolled male athletes, with relatively few studies involving female participants, limiting the ability to draw sex-specific conclusions regarding the effects of beetroot-derived nitrate supplementation. Variations in training status, competitive level, physiological characteristics, and habitual dietary nitrate intake may have further contributed to the inconsistent responses reported across studies. This review was restricted to English-language, peer-reviewed publications, which may have introduced language and publication bias. Furthermore, as a scoping review, the objective was to map and summarize the existing evidence rather than assess methodological quality or generate pooled effect estimates. Consequently, the findings provide an overview of the current evidence base rather than definitive conclusions regarding the effectiveness of beetroot-derived nitrate supplementation in highly trained and elite athletes. Finally, the included studies comprised both acute and repeated beetroot-derived nitrate supplementation protocols, which may produce different physiological and performance responses. Future evidence syntheses should consider evaluating these supplementation strategies separately.

Future research should prioritize adequately powered, multi-center randomized controlled trials with a greater representation of female athletes and standardized supplementation protocols. Further investigation is needed to establish optimal dosing strategies, supplementation duration, and timing across different sporting disciplines. Studies should also explore factors influencing individual responsiveness, including training status, oral microbiome composition, habitual dietary nitrate intake, and baseline nitric oxide availability. Finally, longitudinal and mechanistic research conducted in real-world competitive settings is warranted to clarify the long-term effects of beetroot-derived nitrate supplementation on performance, recovery, and physiological adaptation in elite sporting populations.

## 4. Conclusions

This scoping review explored the influence of beetroot-derived nitrate supplementation on performance, recovery, and physiological outcomes in elite athletes. A total of 31 studies were included, and their characteristics indicate that they were conducted across a wide range of sports worldwide and that most selected men as subjects. The training characteristics indicate that the selected studies represent a wide spectrum of sports disciplines, including endurance sports and intermittent team sports. The study’s findings highlight that, among the selected elite athletes, beetroot-derived nitrate supplementation may confer a modest ergogenic benefit for certain performance and physiological outcomes. The beneficial effects were more pronounced with intermittent high-intensity exercise, anaerobic power, and certain recovery markers, whereas endurance performance benefits remain inconsistent. The discrepancies in outcomes across studies result from heterogeneity in supplementation doses, training strategies, and the individual characteristics of participating athletes. Future research should focus on individualized supplementation strategies, dose–response relationships, and long-term adaptations in elite sporting populations.

## Figures and Tables

**Table 1:** Inclusion criteria and exclusion criteria.

Domain	Inclusion Criteria	Exclusion Criteria
Population	Studies including athletes classified as McKay Tier 3–5 (highly trained, elite, or world-class), determined by explicit evidence of competitive level (e.g., national or international participation, rankings, or professional status).	Studies including Tier 1–2 (trained/recreational/untrained) participants; mixed samples where Tier 3–5 data are not separately extractable; studies with unclear or unreported athlete level.
Concept (Intervention)	Beetroot-derived nitrate supplementation as a standalone intervention (e.g., juice, concentrate, powder, extract, whole food), administered acutely or chronically, and compared with a placebo or control condition.	Non-beetroot nitrate sources (e.g., sodium/potassium nitrate); multi-ingredient interventions where the independent effect of beetroot cannot be isolated; studies without a comparator.
Outcomes	At least one exercise-related outcome, including: (i) performance (e.g., time trial, sprint, power), (ii) recovery (e.g., fatigue, muscle damage), or (iii) physiological/biomarker responses (e.g., VO_2_, lactate, RPE).	Studies reporting only non-exercise outcomes (e.g., clinical, cognitive, or resting-only measures) without an exercise component.
Context	Studies conducted in any sport or exercise-related setting, including laboratory-based testing, field-based assessments, training sessions, or applied performance environments.	Studies conducted in non-exercise contexts (e.g., purely clinical or resting conditions without exercise testing).
Study Design	Randomized controlled trials (RCTs), including cross-over and parallel designs, with a placebo or control comparator.	Non-randomized studies, observational designs, case reports, reviews, editorials, conference abstracts without full data, and protocols without results.
Reporting/Duplication	Original studies with extractable data; secondary analyses included only if they report additional relevant outcomes.	Duplicate publications or secondary reports without new data.
Language/Timeframe	Studies published in English, with no restriction on publication date.	Non-English studies were excluded due to feasibility constraints.

## Data Availability

No new data were created or analyzed in this study. Data sharing is not applicable to this article.

## Supplementary Materials

The following supporting information can be downloaded at: https://www.mdpi.com/article/10.3390/nu18152551/s1, File S1: Preferred Reporting Items for Systematic Reviews and Meta-Analyses extension for Scoping Reviews (PRISMA-ScR) Checklist; File S2: Search Strategy.
