# Supplementary material for: Effects of Beetroot-Derived Nitrate Supplementation in Highly Trained and Elite Athletes: A Scoping Review of Randomized Controlled Trials on Performance, Recovery, and Physiological Outcomes
Source: Nutrients. 2026 Aug 4;18(15):2551. doi: 10.3390/nu18152551 (PMC13468440; doi:10.3390/nu18152551)
Supplement: Supplementary file 1 [file nutrients-18-02551-s001.zip › Supplementary File S2.pdf]

Effects of Beetroot-Derived Nitrate Supplementation in Highly Trained and Elite Athletes: A Scoping Review Protocol of Randomized Controlled Trials on Performance, Recovery, and Physiological Outcomes

---

Search Strategy

Scopus = 428

(TITLE-ABS-KEY ((beetroot* OR "beta vulgaris")) AND TITLE-ABS-KEY ((athlete* OR sport* OR player* OR exercise* OR "physical activity" OR "Acrobatic Gymnastic*" OR "Alpine Skiing*" OR archery* OR "Artistic Gymnastic*" OR "Artistic Swimming*" OR athletic* OR badminton* OR baseball* OR softball* OR basketball* OR "Beach Handball*" OR "Beach Volleyball*" OR biathlon* OR bobsleigh* OR boxing* OR "Canoe Slalom*" OR "Canoe Sprint*" OR cricket* OR "Cross-Country Skiing*" OR curling* OR "Cycling BMX Freestyle*" OR "Cycling BMX Racing*" OR "Cycling Mountain Bike*" OR "Cycling Road*" OR "Cycling Track*" OR diving* OR equestrian* OR fencing* OR "Figure Skating*" OR football* OR "Freestyle Skiing*" OR futsal* OR golf* OR handball* OR hockey* OR "Ice Hockey*" OR judo* OR karate* OR "Lacrosse*" OR luge* OR "Marathon Swimming*" OR "Modern Pentathlon*" OR "Nordic Combined*" OR "Rhythmic Gymnastic*" OR "Roller Speed Skating*" OR rowing* OR "Rugby Sevens*" OR sailing* OR shooting* OR "Short Track Speed Skating*" OR "Skateboarding*" OR "Ski Jumping*" OR "Ski Mountaineering*" OR snowboard* OR "Speed Skating*" OR "Sport Climbing*" OR squash* OR surfing* OR swimming* OR taekwondo* OR tennis* OR trampoline* OR triathlon* OR volleyball* OR "Water Polo*" OR weightlifting* OR wrestling* OR "skeleton bobsled" OR "Track and field" OR running* OR sprint* OR jumping OR throwing OR racewalking OR "cross country" OR "cross-country" OR hurdles OR "steeplechase" OR marathon OR "Half-marathon" OR "Half marathon" OR "Pole vault" OR jump* OR "High jump" OR "Long jump" OR "Triple jump" OR "Discus throw" OR "Hammer throw" OR "Javelin throw" OR "Pentathlon" OR "Heptathlon" OR "Decathlon" OR boxer* OR wrestler* OR karateka* OR fencer* OR gymnast* OR runner* OR sprinter* OR racewalker* OR "cross country runner*" OR "cross-country runner*" OR hurdler* OR "steeplechase runner*" OR "marathon runner*" OR "half-marathon runner*" OR "pole vaulter*" OR jumper* OR "high jumper*" OR "long jumper*" OR "triple jumper*" OR "discus thrower*" OR "hammer thrower*" OR "javelin thrower*" OR pentathlete* OR heptathlete* OR decathlete* OR swimmer* OR diver* OR cyclist* OR skier* OR snowboarder* OR "skater*" OR biathlete* OR luger* OR curler* OR archer* OR shooter* OR rower* OR canoeist* OR "canoe slalom paddler*" OR "canoe sprint paddler*" OR triathlete* OR weightlifter* OR climber* OR "sport climber*" OR skateboarder* OR surfer* OR "roller speed skater*" OR equestrian*)) AND TITLE-ABS-KEY (("randomized controlled trial*" OR "randomised controlled trial*" OR "controlled clinical trial*" OR randomized* OR randomised OR RCT)))

PubMed = 321

("beetroot*"[Title/Abstract] OR "Beta vulgaris"[Title/Abstract] OR "Beta vulgaris"[MeSH Terms]) AND ("Athletes"[MeSH Terms] OR "Exercise"[MeSH Terms] OR "Motor Activity"[MeSH Terms] OR "Sports"[MeSH Terms] OR "athlete*"[Title/Abstract] OR "sport*"[Title/Abstract] OR "player*"[Title/Abstract] OR "exercise*"[Title/Abstract] OR "physical activity"[Title/Abstract] OR "acrobatic gymnastic*"[Title/Abstract] OR "alpine skiing*"[Title/Abstract] OR "archery*"[Title/Abstract] OR "artistic gymnastic*"[Title/Abstract] OR "artistic swimming*"[Title/Abstract] OR "athletic*"[Title/Abstract] OR "badminton*"[Title/Abstract] OR "baseball*"[Title/Abstract]

OR "softball*"[Title/Abstract] OR "basketball*"[Title/Abstract] OR "beach handball*"[Title/Abstract] OR "beach volleyball*"[Title/Abstract] OR "biathlon*"[Title/Abstract] OR "bobsleigh*"[Title/Abstract] OR "boxing*"[Title/Abstract] OR "canoe slalom*"[Title/Abstract] OR "canoe sprint*"[Title/Abstract] OR "cricket*"[Title/Abstract] OR "cross country skiing*"[Title/Abstract] OR "curling*"[Title/Abstract] OR "cycling road*"[Title/Abstract] OR "cycling track*"[Title/Abstract] OR "diving*"[Title/Abstract] OR "equestrian*"[Title/Abstract] OR "fencing*"[Title/Abstract] OR "figure skating*"[Title/Abstract] OR "football*"[Title/Abstract] OR "freestyle skiing*"[Title/Abstract] OR "futsal*"[Title/Abstract] OR "golf*"[Title/Abstract] OR "handball*"[Title/Abstract] OR "hockey*"[Title/Abstract] OR "ice hockey*"[Title/Abstract] OR "judo*"[Title/Abstract] OR "karate*"[Title/Abstract] OR "lacrosse*"[Title/Abstract] OR "luge*"[Title/Abstract] OR "marathon swimming*"[Title/Abstract] OR "modern pentathlon*"[Title/Abstract] OR "nordic combined*"[Title/Abstract] OR "rhythmic gymnastic*"[Title/Abstract] OR "roller speed skating*"[Title/Abstract] OR "rowing*"[Title/Abstract] OR "rugby sevens*"[Title/Abstract] OR "sailing*"[Title/Abstract] OR "shooting*"[Title/Abstract] OR "short track speed skating*"[Title/Abstract] OR "skateboarding*"[Title/Abstract] OR "ski jumping*"[Title/Abstract] OR "ski mountaineering*"[Title/Abstract] OR "snowboard*"[Title/Abstract] OR "speed skating*"[Title/Abstract] OR "sport climbing*"[Title/Abstract] OR "squash*"[Title/Abstract] OR "surfing*"[Title/Abstract] OR "swimming*"[Title/Abstract] OR "taekwondo*"[Title/Abstract] OR "tennis*"[Title/Abstract] OR "trampoline*"[Title/Abstract] OR "triathlon*"[Title/Abstract] OR "volleyball*"[Title/Abstract] OR "water polo*"[Title/Abstract] OR "weightlifting*"[Title/Abstract] OR "wrestling*"[Title/Abstract] OR "Track and field"[Title/Abstract] OR "running*"[Title/Abstract] OR "sprint*"[Title/Abstract] OR "jumping"[Title/Abstract] OR "throwing"[Title/Abstract] OR "racewalking"[Title/Abstract] OR "cross-country"[Title/Abstract] OR "cross-country"[Title/Abstract] OR "hurdles"[Title/Abstract] OR "steeplechase"[Title/Abstract] OR "marathon"[Title/Abstract] OR "half marathon"[Title/Abstract] OR "half marathon"[Title/Abstract] OR "Pole vault"[Title/Abstract] OR "jump*"[Title/Abstract] OR "High jump"[Title/Abstract] OR "Long jump"[Title/Abstract] OR "Triple jump"[Title/Abstract] OR "Discus throw"[Title/Abstract] OR "Hammer throw"[Title/Abstract] OR "Javelin throw"[Title/Abstract] OR "pentathlon*"[Title/Abstract] OR "heptathlon*"[Title/Abstract] OR "decathlon*"[Title/Abstract] OR "boxer*"[Title/Abstract] OR "wrestler*"[Title/Abstract] OR "karateka*"[Title/Abstract] OR "fencer*"[Title/Abstract] OR "gymnast*"[Title/Abstract] OR "runner*"[Title/Abstract] OR "sprinter*"[Title/Abstract] OR "racewalker*"[Title/Abstract] OR "cross country runner*"[Title/Abstract] OR "cross country runner*"[Title/Abstract] OR "hurdler*"[Title/Abstract] OR "steeplechase runner*"[Title/Abstract] OR "marathon runner*"[Title/Abstract] OR "half marathon runner*"[Title/Abstract] OR "pole vaulter*"[Title/Abstract] OR "jumper*"[Title/Abstract] OR "high jumper*"[Title/Abstract] OR "long jumper*"[Title/Abstract] OR "triple jumper*"[Title/Abstract] OR "discus thrower*"[Title/Abstract] OR "hammer thrower*"[Title/Abstract] OR "javelin thrower*"[Title/Abstract] OR "pentathlete*"[Title/Abstract] OR "heptathlete*"[Title/Abstract] OR "decathlete*"[Title/Abstract] OR "swimmer*"[Title/Abstract] OR "diver*"[Title/Abstract] OR "cyclist*"[Title/Abstract] OR "skier*"[Title/Abstract] OR "snowboarder*"[Title/Abstract] OR "skater*"[Title/Abstract] OR "biathlete*"[Title/Abstract] OR "luger*"[Title/Abstract] OR "curler*"[Title/Abstract] OR "archer*"[Title/Abstract] OR "shooter*"[Title/Abstract] OR "rower*"[Title/Abstract] OR

"canoeist*"[Title/Abstract] OR "triathlete*"[Title/Abstract] OR "weightlifter*"[Title/Abstract] OR "climber*"[Title/Abstract] OR "sport climber*"[Title/Abstract] OR "skateboarder*"[Title/Abstract] OR "surfer*"[Title/Abstract]) AND ("Randomized Controlled Trial"[Publication Type] OR "Controlled Clinical Trial"[Publication Type] OR "randomized"[Title/Abstract] OR "randomised"[Title/Abstract] OR "RCT"[Title/Abstract])

Web of Science = 353

TS=((beetroot* OR "beta vulgaris")) AND TS=((athlete* OR sport* OR player* OR exercise* OR "physical activity" OR "Acrobatic Gymnastic*" OR "Alpine Skiing*" OR archery* OR "Artistic Gymnastic*" OR "Artistic Swimming*" OR athletic* OR badminton* OR baseball* OR softball* OR basketball* OR "Beach Handball*" OR "Beach Volleyball*" OR biathlon* OR bobsleigh* OR boxing* OR "Canoe Slalom*" OR "Canoe Sprint*" OR cricket* OR "Cross-Country Skiing*" OR curling* OR "Cycling BMX Freestyle*" OR "Cycling BMX Racing*" OR "Cycling Mountain Bike*" OR "Cycling Road*" OR "Cycling Track*" OR diving* OR equestrian* OR fencing* OR "Figure Skating*" OR football* OR "Freestyle Skiing*" OR futsal* OR golf* OR handball* OR hockey* OR "Ice Hockey*" OR judo* OR karate* OR "Lacrosse*" OR luge* OR "Marathon Swimming*" OR "Modern Pentathlon*" OR "Nordic Combined*" OR "Rhythmic Gymnastic*" OR "Roller Speed Skating*" OR rowing* OR "Rugby Sevens*" OR sailing* OR shooting* OR "Short Track Speed Skating*" OR "Skateboarding*" OR "Ski Jumping*" OR "Ski Mountaineering*" OR snowboard* OR "Speed Skating*" OR "Sport Climbing*" OR squash* OR surfing* OR swimming* OR taekwondo* OR tennis* OR trampoline* OR triathlon* OR volleyball* OR "Water Polo*" OR weightlifting* OR wrestling* OR "skeleton bobsled" OR "Track and field" OR running* OR sprint* OR jumping OR throwing OR ropewalking OR "cross country" OR "cross-country" OR hurdles OR "steeplechase" OR marathon OR "Half-marathon" OR "Half marathon" OR "Pole vault" OR jump* OR "High jump" OR "Long jump" OR "Triple jump" OR "Discus throw" OR "Hammer throw" OR "Javelin throw" OR "Pentathlon" OR "heptachlor" OR "Decathlon" OR boxer* OR wrestler* OR karateka* OR fencer* OR gymnast* OR runner* OR sprinter* OR racewalker* OR "cross country runner*" OR "cross-country runner*" OR hurdler* OR "steeplechase runner*" OR "marathon runner*" OR "half-marathon runner*" OR "pole vaulter*" OR jumper* OR "high jumper*" OR "long jumper*" OR "triple jumper*" OR "discus thrower*" OR "hammer thrower*" OR "javelin thrower*" OR pentathlete* OR heptathlete* OR decathlete* OR swimmer* OR diver* OR cyclist* OR skier* OR snowboarder* OR "skater*" OR biathlete* OR luger* OR curler* OR archer* OR shooter* OR rower* OR canoeist* OR "canoe slalom paddler*" OR "canoe sprint paddler*" OR triathlete* OR weightlifter* OR climber* OR "sport climber*" OR skateboarder* OR surfer* OR "roller speed skater*" OR equestrian*)) AND TS=(("randomized controlled trial*" OR "randomised controlled trial*" OR "controlled clinical trial*" OR randomized* OR randomised OR RCT))

https://www.webofscience.com/wos/woscc/summary/cd8809b5-c95f-4227-8d3f-221d915cc020-01b3590af1/relevance/1
